# Simultaneous LC-MS/MS determination of diacylhydrazine ecdysone receptor agonist insecticides in tomatoes: field dissipation, household washing effects, and dietary risk assessment

**DOI:** 10.1039/d5ra08001k

**Published:** 2025-12-01

**Authors:** Rania M. Abd El-Hamid, Nevein S. Ahmed, Hanim M. Soliman, Fahad S. Almulhim, Osama I. Abdallah

**Affiliations:** a Department of Pesticide Residues and Environmental Pollution, Central Agricultural Pesticide Laboratory, Agricultural Research Center Giza 12618 Egypt shebin_osama@yahoo.com; b Department of Food Chemistry, Food Safety Laboratory Qassim Municipality, Qassim Region Buraydah 52571 Saudi Arabia

## Abstract

Tomato (*Lycopersicon esculentum* Mill.) is among the most widely cultivated vegetables worldwide, yet its production is vulnerable to insect pests, necessitating extensive insecticide use. In this study, a sensitive and robust LC-MS/MS method was developed and validated for the simultaneous determination of four insecticides—methoxyfenozide, tebufenozide, chromafenozide, and halofenozide—in tomato fruits. The method demonstrated excellent linearity (*R*^2^ > 0.99), LOQ = 0.01 mg kg^−1^ for all analytes, mean recoveries 81.2–97.6%, and matrix effects between −18.7% and −4.7%, confirmed its reliability for routine residue surveillance. Given the global reliance on methoxyfenozide and tebufenozide, field dissipation studies were conducted using recommended and double recommended doses under open-field conditions in Egypt to assess their dissipation behavior, pre-harvest intervals (PHIs), and associated dietary risks. To support terminal-residue evaluation at harvest, samples were collected at 1, 3, and 7 days after the final application, coinciding with commercial harvest maturity. Both compounds followed first-order kinetics, with rapid dissipation reflected in half-lives ranging from 1.99 to 2.31 days and PHIs between 1.32 and 4.36 days, ensuring compliance with international maximum residue limits. Chronic dietary exposure, expressed as %ADI (RQ = NEDI/ADI × 100), was low: the maximum across all scenarios was 4.06% for methoxyfenozide and 39.44% for tebufenozide; thus, NEDI values were <100% of ADI in every case, indicating no chronic health concern. Furthermore, household processing methods—particularly washing with 5% acetic acid—significantly reduced residues by up to 83.87%, offering an accessible strategy for minimizing consumer exposure. These findings provide critical evidence to support the safe and sustainable use of insect growth regulators in tomato production while underscoring the value of simple post-harvest interventions to enhance food safety.

## Abstract 2

Tomato (*Lycopersicon esculentum* Mill.) is among the most widely cultivated vegetables worldwide, yet its production is vulnerable to insect pests, necessitating extensive insecticide use.

## Graphical abstract

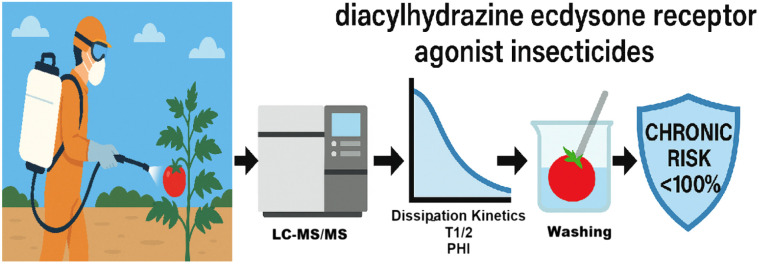

## 1. Introduction

Tomato (*Solanum lycopersicum* L.) is among the most widely cultivated and consumed vegetables worldwide, ranking second to potato (*Solanum tuberosum* L.) in terms of production and economic importance.^1^ In Egypt, tomatoes underpin the vegetable sector—approximately one-quarter of annual vegetable acreage—with newly reclaimed lands offering scope to improve yields and fruit quality.^2^ Their dietary value matches this agronomic prominence: tomatoes are recognized as functional foods that supply lycopene, tomatine, antioxidants, calcium, niacin, and vitamins A, C, and E—bioactives associated with reduced risks of certain cancers and other chronic diseases.^3–5^

Tomato productivity remains constrained by insect pests—particularly endophytic or other tissue-dwelling species that escape broad-spectrum contact sprays. Any brief suppression is offset by selection for resistance and residues that threaten health and degrade fruit quality.^6,7^ Since the late 1980s, diacylhydrazine ecdysone receptor agonists (IRAC Group 18) have provided a selective alternative to broad-spectrum contact insecticides, acting by mimicking the moulting hormone to trigger precocious molts in target Lepidopte^8^

First commercialized by Rohm & Haas, methoxyfenozide and tebufenozide (Fig. S1a and b) provide selective control of lepidopteran larvae, including populations resistant to pyrethroids and organophosphates.^9,10^ As 20-hydroxyecdysone mimics, they trigger precocious, incomplete ecdysis leading to larval death while limiting non-target effects. In tomato, methoxyfenozide controls *Spodoptera* spp. with strong translaminar activity, whereas tebufenozide is most effective against early instars of *Helicoverpa armigera* and *Spodoptera littoralis* and is compatible with biological control.^9,10^ Being largely non-systemic and ingestion-activated, these actives fit integrated pest management, but rigorous residue monitoring remains essential.

LC-MS/MS dissipation has been reported for methoxyfenozide,^11,12^ tebufenozide,^13^ and chromafenozide (Fig. S1c)^14^ in non-tomato matrices. However, tomato-specific data are limited, and no validated method simultaneously quantifies methoxyfenozide, tebufenozide, chromafenozide, and halofenozide (Fig. S1d) in a single tomato matrix. Under Egypt's hot-arid, open-field conditions, dissipation kinetics and terminal residues of methoxyfenozide and tebufenozide remain poorly characterized, hindering preharvest-interval (PHI) setting and dietary risk assessment.

Household washing can reduce residues.^15,16^ However, standardized, tomato-specific evaluations especially for methoxyfenozide and tebufenozide are limited, constraining evidence-based consumer guidance.^17^

Accordingly, we (i) develop and validate a unified, optimized LC-MS/MS method for simultaneous quantification of the diacylhydrazines methoxyfenozide, tebufenozide, chromafenozide, and halofenozide in tomatoes; (ii) characterize open-field dissipation kinetics, terminal residues, and chronic dietary risk for methoxyfenozide and tebufenozide under Egyptian conditions; and (iii) benchmark household-washing protocols (tap water, acetic acid, sodium bicarbonate) to produce tomato-specific, statistically supported residue-mitigation guidance.

## 2. Materials and methods

### 2.1. Chemicals and standard preparations

Reference standards of methoxyfenozide (99.5%), tebufenozide (99.5%), chromafenozide (≥98.5%), and halofenozide (98%) were obtained from Chem Service (West Chester, PA, USA). The commercial formulations used in the field trials—methoxyfenozide 24% SC and tebufenozide 20% SC—were purchased from the local Egyptian market. LC-MS-grade acetonitrile, methanol, ammonium formate, formic acid, and glacial acetic acid were from Fisher Scientific (Waltham, MA, USA). Ultrapure water (18.2 MΩ cm) was produced with an Ultra Clear system (Evoqua Water Technologies, Günzburg, Germany). Anhydrous magnesium sulfate (MgSO_4_) and sodium acetate (CH_3_COONa) were from Chem-Lab NV (Zedelgem, Belgium). Primary–secondary amine (PSA) sorbent was from Agilent Technologies (Santa Clara, CA, USA); multi-walled carbon nanotubes (MWCNTs) from Shilpent (Maharashtra, India); and sodium bicarbonate (NaHCO_3_) from Loba Chemie (Mumbai, India).

Individual stock solutions (1000 µg mL^−1^ per analyte) were prepared in acetonitrile and stored at −20 °C. Intermediate mixed standards (100 µg mL^−1^ per analyte) were prepared by dilution in acetonitrile, and daily working mixtures (10 µg mL^−1^ per analyte) were prepared by serial dilution on the day of analysis. Matrix-matched calibration solutions were prepared by spiking blank tomato extracts.

### 2.2. Field experiment

#### 2.2.1. Dissipation study

The field trial was conducted in summer 2024 at El-Salheya El-Gedida city, Sharqia Governorate, Egypt, using a randomized block design with three replicates per treatment; each plot measured 10 × 15 m. The crop was a fresh-market F1 hybrid tomato. Applications were made 84 days after transplanting, when fruits had reached full ripeness (complete red color suitable for commercial harvest). Methoxyfenozide 24% SC (90/180 g a.i. ha^−1^) and tebufenozide 20% SC (150/300 g a.i. ha^−1^) were applied at 1000 L ha^−1^ with a 20 L backpack sprayer; products were applied separately and tanks/lines triple-rinsed between treatments (label and 2× label rates). Meteorological conditions during 0–21 days after treatment (DAT) were: mean daily maximum 35.8 °C (33.1–39.2 °C), minimum 23.9 °C (21.8–26.5 °C), mean relative humidity 44% (34–58%), mean 2 m wind speed 1.6 m s^−1^ (0.8–2.7 m s^−1^), with no rainfall. For dissipation profiling, composite tomato samples were collected at 0 (2 h post-application), 1, 3, 5, 7, 10, 14 and 21 days, alongside untreated-plot controls. Sample handling and storage followed the general procedure in Section 2.3.

#### 2.2.2. Terminal residues

The terminal-residue trial was conducted in separate randomized block design (RBD) plots (three replicates per treatment; 10 × 15 m each). Methoxyfenozide 24% SC (90 and 180 g a.i. ha^−1^) and tebufenozide 20% SC (150 and 300 g a.i. ha^−1^) were applied at label and 2× label rates in two spray programs: two applications and three applications, each spaced 10 days apart. The programs were initiated 84 days after transplanting, when fruits were at full ripeness, suitable for commercial harvest. To represent harvest conditions, composite tomato samples were collected at 1, 3, and 7 days after the final treatment, coinciding with commercial harvest maturity for the study variety under the site's agronomic conditions. At each time point, treated-plot samples were collected alongside untreated control samples from separate plots. Sample handling and storage followed Section 2.3. Terminal-residue measurements were used as inputs for chronic dietary exposure (NEDI) and risk quotient (RQ = NEDI/ADI × 100) calculations as described in Section 2.7, and to support PHI interpretation (Section 2.8).

#### 2.2.3. Sample collection and handling

At each sampling time, a composite tomato sample of ≥1.0 kg per replicate was collected and transported from the field in insulated coolers with ice packs. Samples were cut into 2–3 cm pieces, pre-frozen overnight at −20 °C, and homogenized to a uniform slurry using a Hobart food cutter (Hobart Corp., Troy, OH, USA). Homogenates were portioned into pre-labelled polypropylene tubes and stored at −20 °C until analysis.

### 2.3. Extraction and cleanup

A 10.0 ± 0.1 g portion of homogenized frozen tomato was weighed into a 50 mL polypropylene centrifuge tube. Acetonitrile acidified with 1% (v/v) glacial acetic acid (10.0 mL) was added, the tube capped, and the mixture vortex-mixed for 2 min. QuEChERS acetate-buffering salts (4.0 g anhydrous MgSO_4_ and 1.0 g sodium acetate, CH_3_COONa) were added.^18^ The tube was immediately shaken vigorously for 1 min to prevent salt agglomeration and then centrifuged at 5000 rpm for 5 min at room temperature. An aliquot (2.0 mL) of the supernatant was transferred to a 15 mL centrifuge tube preloaded with dispersive-SPE (d-SPE) sorbents (per 2.0 mL extract: 50 mg PSA, 5 mg MWCNTs, 300 mg MgSO_4_), vortex-mixed for 1 min, and centrifuged for 5 min at 5000 rpm. A 1.0 mL portion of the cleaned extract was filtered through a 0.22 µm nylon syringe filter into LC-MS/MS vials. When residue concentrations exceeded the calibration range, extracts were diluted with blank tomato matrix extract prepared identically to samples; the dilution factor was recorded and applied during quantification.

### 2.4. LC-MS/MS

Chromatographic separation was performed on a Dionex Ultimate 3000 RS UHPLC (Thermo Fisher Scientific, USA) fitted with an ACQUITY UPLC BEH C18 column (1.7 µm, 2.1 × 100 mm; Waters, Ireland) held at 40 °C. The mobile phases were A: water with 5 mM ammonium formate and 0.1% formic acid, and B: methanol: water 98 : 2 v/v with 5 mM ammonium formate and 0.1% formic acid, delivered at 0.30 mL min^−1^. The gradient was 2% B (0–1 min) → linear ramp to 98% B (5 min) → hold to 10 min → return to 2% B (10.1 min) → re-equilibrate to 17 min. The injection volume was 2 µL.

Mass spectrometric detection utilized a Thermo Fisher Scientific triple-quadrupole mass spectrometer (TSQ Altis) (Thermo Fisher Scientific, USA) equipped with a heated electrospray ionization (H-ESI) source. Ionization was performed in positive mode for methoxyfenozide, tebufenozide, and chromafenozide, and in negative mode for halofenozide, with fast polarity switching in a single run. Data were acquired in multiple reaction monitoring (MRM) with two transitions per analyte (quantifier/qualifier); precursor → product ions, collision energies, RF lens voltages, and retention times are listed in Table 1. Source/interface conditions were optimized by direct infusion using a Fusion F100T2 digital dual syringe pump (Chemyx, Stafford, TX, USA). Data acquisition and processing were performed in Trace Finder v4.1 (Thermo Fisher Scientific, USA).

**Table 1:** MS/MS parameters for the determination of chromafenozide, halofenozide, methoxyfenozide, and tebufenozide

Compound	Retention time (min)	Polarity	Precursor ion (*m*/*z*)	Product ion (*m*/*z*)	Collision energy (V)	RF lens (V)
Methoxyfenozide	8.90	Positive	369.1	149.0	17	41
				313.1	10	41
Tebufenozide	9.22	Positive	353.2	132.9	19	62
				297.0	10	62
Chromafenozide	9.04	Positive	395.1	146.9	44	66
				174.9	15	66
Halofenozide	8.76	Negative	329.0	120.9	19	69
				77.0	33	69

### 2.5. Method validation

Method validation followed SANTE/11312/2021 for the four diacylhydrazine ecdysone-receptor agonists (methoxyfenozide, tebufenozide, chromafenozide, halofenozide) in tomatoes.^19^ Linearity was established using matrix-matched calibration at eight levels (0.001–0.25 mg kg^−1^), fitted by weighted linear regression (1/*x*). Linearity was assessed using matrix-matched calibration at eight concentration levels (1–200 µg kg^−1^), fitted with weighted (1/*x*) linear regression. Calibration performance was evaluated from the regression coefficient (*R*^2^), back-calculated residuals, and acceptance criteria were *R*^2^ ≥ 0.99 and absolute residuals ≤ 20% across the range. Sensitivity was defined operationally at S/N ≥ 3 (limit of detection, LOD) and S/N ≥ 10 (limit of quantitation, LOQ), with performance-based LOQs confirmed in tomato by meeting SANTE trueness/precision criteria at the LOQ. Accuracy (trueness) was assessed by recoveries at 0.01, 0.10, and 1.0 mg kg^−1^ (*n* = 3 per level) with acceptance 70–120%. Precision was evaluated as intra-day repeatability (RSD_r_; six spikes in one day) and inter-day repeatability (RSD_R_; three consecutive days at the LOQ), with acceptance ≤20% RSD. Matrix effects (ME) were quantified from slope ratios of post-extraction spiked matrix *vs.* solvent standards: ME% = [(slope_matrix/slope_solvent) − 1] × 100, where negative values indicate signal suppression and positive values signal enhancement; effects were mitigated using matrix-matched calibration.^20^ A compound was positively identified only when its retention time matched that of matrix-matched standards within ±0.1 min, both MRM transitions were present, the qualifier/quantifier ion ratio was within SANTE tolerances of 30%, and no corresponding signal occurred in solvent or matrix blanks.

### 2.6. Washing treatment

To evaluate household washing efficacy, tomato fruits were collected one day after application from treated plots and randomly assigned to five regimens (three replicates per regimen; 10 fruits per replicate): (i) tap water immersion (pH 7.9, total dissolved solids 508 mg L^−1^, free chlorine 0.24 mg L^−1^), (ii) 2% (v/v) acetic acid, (iii) 5% (v/v) acetic acid, (iv) 2% (w/v) sodium bicarbonate (NaHCO_3_), and (v) 5% (w/v) NaHCO_3_. The fruit-to-solution ratio was standardized at 10 fruits in 5.0 L (0.5 L per fruit). Washes were performed at 23–25 °C with gentle hand agitation for 10 min to simulate household practice. After treatment, fruits were washed under tape water for 10 seconds and air-dried on clean, absorbent paper towels.

Two controls were included: (i) an unwashed control—raw fruit collected from the treated plots at the same sampling times to benchmark washing efficacy—and (ii) an unsprayed field control from separate untreated plots to provide matrix blanks. Samples were cut, homogenized, and stored, followed by Section 2.3; LC-MS/MS analysis followed Sections 3 and 4.

### 2.7. Risk assessment

Chronic dietary exposure was estimated as the National Estimated Daily Intake (NEDI) (eqn (1)):1
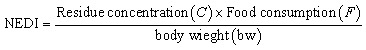
where *C* is the measured residue concentration across the relevant sampling intervals in tomatoes (mg kg^−1^), *F* is daily tomato consumption (0.2009 kg per person per day), and bw is body weight (60 kg).^21^ Chronic risk was expressed as the Risk Quotient (*RQ*, %) (eqn (2)):

2 RQ% = (NEDI/ADI) × 100

Acceptable Daily Intake (ADI) values were 0.02 mg kg per bw per day for tebufenozide,^22^ and 0.10 mg kg per bw per day for methoxyfenozide.^23^ Consistent with international guidance, RQ < 100% indicates acceptable chronic dietary risk.^24,25^

### 2.8. Calculations and statistical analysis

Residue dissipation for methoxyfenozide and tebufenozide was described by a first-order model using eqn (3).^26^

3 *C*_*t*_ = *C*_*0*_ × e^−*kt*^

where *C*_*t*_ is the residue concentration at time *t* (days), *C*_0_ is the fitted initial concentration, and *k* is the dissipation rate constant (per day).

The dissipation half-life and the pre-harvest interval (PHI) to reach a specified maximum residue limit (MRL) were calculated using eqn (4) and (5).^27,28^4
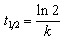
5
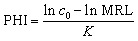


Residues in washed groups were statistically compared with the paired unwashed controls using one-way ANOVA followed by Tukey's HSD (*α* = 0.05). Model fitting and parameter calculations were performed in Microsoft Excel (version 2024).

## 3. Results and discussion

### 3.1. LC-MS/MS optimization

MS/MS parameters for methoxyfenozide, tebufenozide, chromafenozide, and halofenozide were optimized using full-scan acquisition (*m*/*z* 100–500) followed by product-ion scans of a mixed standard solution. Full-scan spectra enabled selection of the precursor ions at *m*/*z* 369.1 (methoxyfenozide), 353.2 (tebufenozide), 395.1 (chromafenozide), and 329.0 (halofenozide) (Fig. S2). Electrospray ionization (ESI) was operated in positive mode for methoxyfenozide, tebufenozide, and chromafenozide, whereas halofenozide exhibited a higher response in negative mode.

After precursor selection, product-ion scans were acquired while automatically varying the collision energy (CE) between 0 to ±200 V to identify the most intense and stable fragments (Fig. S2). Three candidate fragments were initially evaluated per analyte; two transitions were retained on the basis of intensity and repeatability. In line with European Commission Decision 2002/657/EC,^29^ the most intense transition was used for quantification and the second for confirmation.

The selected MRM transitions were: 369.1 → 149.0/313.1 (methoxyfenozide), 353.2 → 132.9/297.0 (tebufenozide), *m*/*z* 395.1 → 146.9/174.9 (chromafenozide), and 329.0 → 120.9/77.0 (halofenozide). Optimized CEs ranged from 10 to 44 V. The full set of MS/MS parameters (precursors, products, CE, RF lens, and retention times) is summarized in Table 1.

Representative MRM chromatograms of spiked samples (Fig. S3) showed baseline separation without interfering peaks. The retention times were 8.90 min (methoxyfenozide), 9.22 min (tebufenozide), 9.04 min (chromafenozide), and 8.76 min (halofenozide), demonstrating sensitive and selective detection with excellent chromatographic resolution and reliable quantification of all target analytes.

### 3.2. Method validation

The analytical method was validated in accordance with SANTE/11312/2021, evaluating linearity, sensitivity, accuracy, precision, and matrix effects for pesticide residue analysis.^19^ Summary results for methoxyfenozide, tebufenozide, chromafenozide, and halofenozide are provided in Tables 2 and 3.

**Table 2:** Validation results

Parameter	Methoxyfenozide	Tebufenozide	Chromafenozide	Halofenozide
Linearity range (mg kg^−1^)	0.005–0.1	0.001–0.25	0.0025–0.1	0.001–0.1
*R* ^2^	0.9987	0.9982	0.9993	0.9996
Residuals (%error)	≤13	≤17	≤14	≤9
Matrix effect (%)	−4.7	−10.2	−18.7	−8.7
LOD (mg kg^−1^)	0.0006	0.0003	0.0008	0.0002
LOQ (mg kg^−1^)	0.01	0.01	0.01	0.01
Intra-day precision (RSD_r_, %) (*n* = 6)	7.1	4.6	11.8	5.8
Inter-day precision (RSD_R_, %) (*n* = 18)	13.3	9.7	16.7	8.8

**Table 3:** Recoveries (%, ±RSD, *n* = 3) for four diacylhydrazine insecticides in fortified tomatoes

Spiking level (mg kg^−1^)	Recovery (% ± RSD) (*n* = 3)			
	Methoxyfenozide	Tebufenozide	Chromafenozide	Halofenozide
0.01	94.1 ± 5.2	90.6 ± 2.8	81.2 ± 7.9	92.5 ± 3.7
0.1	97.6 ± 3.2	92.7 ± 5.1	86.7 ± 2.2	94.7 ± 3.1
1.0	93.8 ± 4.1	94.5 ± 2.9	88.4 ± 3.3	95.5 ± 2.6

#### 3.2.1. Linearity

Excellent linearity was obtained over the working ranges (Fig. S4): methoxyfenozide (0.005–0.25 mg kg^−1^), tebufenozide (0.001–0.25 mg kg^−1^), chromafenozide (0.0025–0.1 mg kg^−1^), and halofenozide (0.001–0.1 mg kg^−1^). The calibration curves yielded *R*^2^ values of 0.9987, 0.9982, 0.9993, and 0.9996, respectively, confirming reliable quantification across the tested intervals. Back-calculated residuals (% error) were uniformly low: methoxyfenozide ≤ 13%, tebufenozide ≤ 17%, chromafenozide ≤ 14%, and halofenozide ≤ 9%.

#### 3.2.2. Sensitivity

Estimated limits of detection (LOD) were 0.0006, 0.0003, 0.0008, and 0.0002 mg kg^−1^ for methoxyfenozide, tebufenozide, chromafenozide, and halofenozide, respectively, evidencing high method sensitivity. A uniform LOQ of 0.01 mg kg^−1^ was established for all analytes; this threshold is at or below the respective MRLs, supporting trace-level monitoring and regulatory compliance. The LOQ met acceptance criteria, with recoveries within 70–120% and precision (RSD) < 20%.

#### 3.2.3. Matrix effects

Matrix effects, calculated from the slope ratio, were −4.7% (methoxyfenozide), −10.2% (tebufenozide), −18.7% (chromafenozide), and −8.7% (halofenozide). All were within ±20%, indicating minimal ion suppression and effective correction *via* matrix-matched calibration.

#### 3.2.4. Precision

Precision was evaluated at the LOQ (0.01 mg kg^−1^). Intra-day repeatability (RSD_r_, *n* = 6) was 7.1% (methoxyfenozide), 4.6% (tebufenozide), 11.8% (chromafenozide), and 5.8% (halofenozide). Inter-day repeatability (RSD_R_, *n* = 18) was 13.3%, 9.7%, 16.7%, and 8.8%, respectively, all within the ≤20% acceptance criterion.

#### 3.2.5. Accuracy

Recoveries determined at 0.01, 0.1, and 1 mg kg^−1^ (each *n* = 3) were 93.8–97.6% for methoxyfenozide; 90.6–94.3% for tebufenozide; 81.2–88.4% for chromafenozide; and 92.5–95.5% for halofenozide. All fell within the 70–120% acceptance window with good precision (RSD ≤ 7.9%) (Table 3).

### 3.3. Dissipation kinetics of methoxyfenozide and tebufenozide in tomatoes

**Fig. 1:**
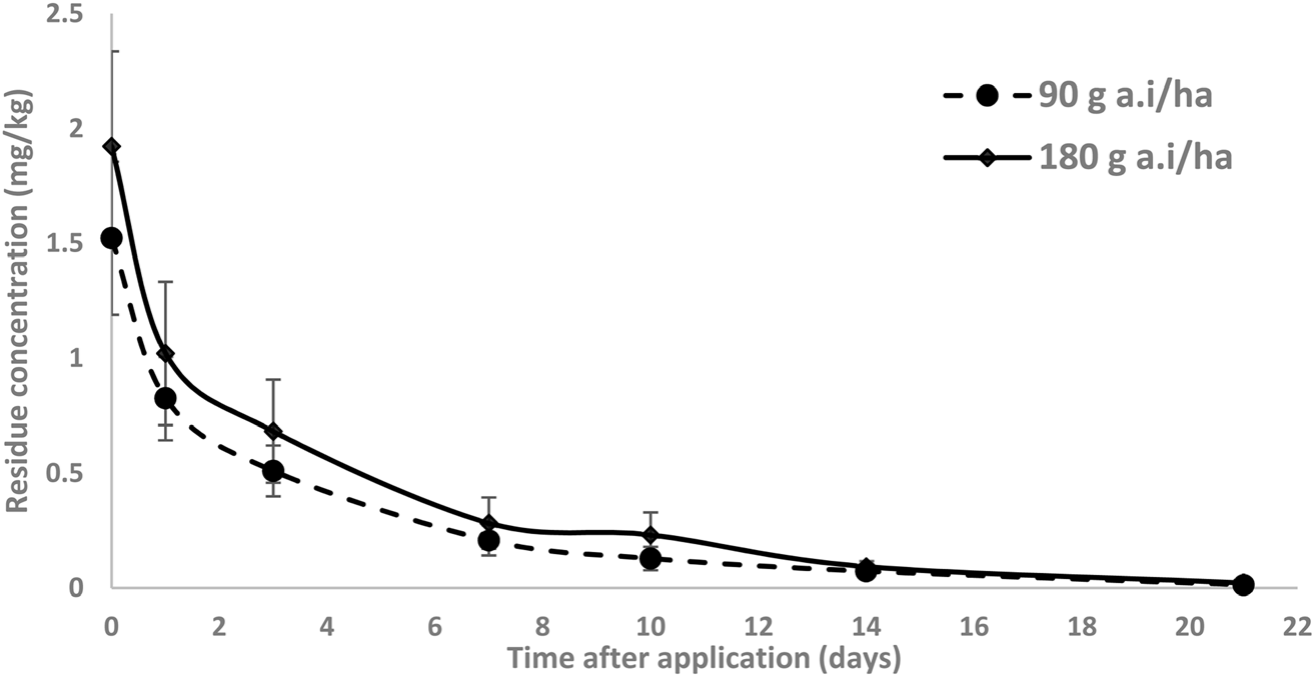
Dissipation of methoxyfenozide residues in tomatoes at two application rates.

**Fig. 2:**
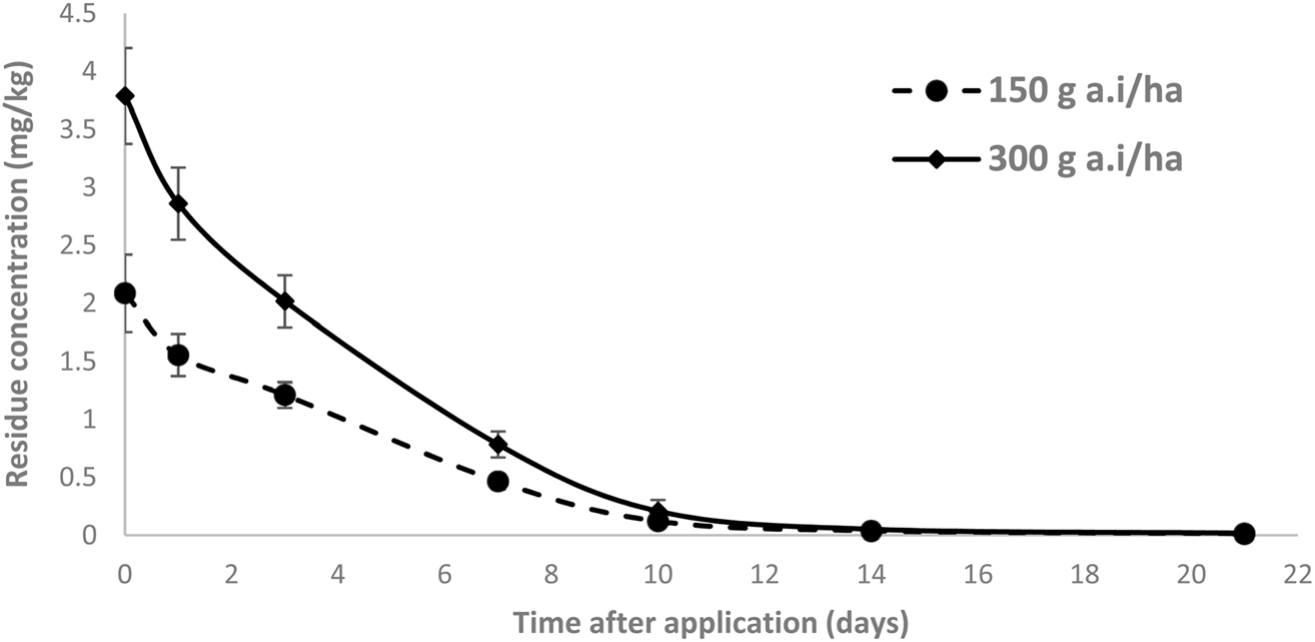
Dissipation of tebufenozide residues in tomatoes at two application rates.

The dissipation of methoxyfenozide (Fig. 1) and tebufenozide (Fig. 2) was studied under open-field conditions at a single dose (T1) and a double dose (T2).

At application rates of 90 and 180 g a.i. ha^−1^ (T1 and T2), initial methoxyfenozide residues were 1.523 and 1.923 mg kg^−1^, respectively. Rapid dissipation followed: by day 1, residues declined to 0.826 (−45.8%) and 1.022 mg kg^−1^ (−46.9%); by day 3, to 0.511 (66.5% dissipation) and 0.683 mg kg^−1^ (64.5%); and by day 7, to 0.208 and 0.283 mg kg^−1^. By day 21, residues were minimized to 0.014 mg kg^−1^ (T1; 99.1%) and 0.022 mg kg^−1^ (T2; 98.9%) (Fig. 1).

Methoxyfenozide dissipation followed first-order kinetics: T1, *C*_*t*_ = 1.101 e^−0.2194*t*^ (*R*^2^ = 0.9822); T2, *C*_*t*_ = 1.424 e^−0.1981*t*^ (*R*^2^ = 0.9849). The corresponding rate constants were *k* = 0.2194 and 0.1981 per day, yielding half-lives of *t*_1/2_ = 3.16 and 3.50 days for T1 and T2, respectively. Using the tomato MRL for methoxyfenozide (0.6 mg kg^−1^; EU MRL database),^30^ the calculated pre-harvest intervals (PHI) were 2.77 days (T1) and 4.36 days (T2) (Table 4).

**Table 4:** Dissipation kinetics of methoxyfenozide and tebufenozide in tomatoes

	Methoxyfenozide		Tebufenozide	
Dose (g a.i. ha^−1^)	90	180	150	300
*R* ^2^	0.9822	0.9849	0.9764	0.9791
*C*_0_ (mg kg^−1^)	1.101	1.424	2.099	3.874
*K* (per day)	0.2194	0.1981	0.2542	0.2713
*t*_1/2_ (days)	3.16	3.5	2.73	2.55
PHI (days)	2.77	4.36	1.32	3.50

Tebufenozide residues were higher at initial deposits—2.088 mg kg^−1^ at 150 g a.i. ha^−1^ (T1) and 3.789 mg kg^−1^ at 300 g a.i. ha^−1^ (T2). Residues declined to 1.556 (T1) and 2.861 mg kg^−1^ (T2) by day 1 (25.46% and 24.49% dissipation), to 1.212 and 2.019 mg kg^−1^ by day 3, and to 0.465 and 0.784 mg kg^−1^ by day 7 (77.74% and 79.31% dissipation). By day 21, only 0.014 mg kg^−1^ (T1; 99.32%) and 0.018 mg kg^−1^ (T2; 99.52%) remained (Fig. 2).

Residue decline for tebufenozide was well captured by a first-order model. Best-fit functions were *C*_*t*_ = 2.099 e^−0.2542*t*^ (T1; *R*^2^ = 0.9764) and *C*_*t*_ = 3.874 e^−0.2713*t*^ (T2; *R*^2^ = 0.9790), corresponding to *k* = 0.2542 and 0.2713 per day. The resulting half-lives were *t*_1/2_ = 2.73 and 2.55 days (T1, T2). Relative to the tomato MRL (1.5 mg kg^−1^; EU MRL database) [30], PHIs were 1.32 days (T1) and 3.50 days (T2) (Table 4).

The methoxyfenozide half-lives (*t*_1/2_ = 3.16–3.50 days) align with Sun *et al.* (2020), who reported *t*_1/2_ = 2.5–3.5 days in cauliflower and approximately 1.2 days in tea.^11^ Likewise, tebufenozide half-lives (*t*_1/2_ = 2.55–2.73 days) are comparable to those reported by Liu *et al.* (2016) for cabbage (*t*_1/2_ = 2.96–4.08 days),^13^ and by Lin *et al.* (2019) for stem lettuce (*t*_1/2_ = 5.0–8.2 days),^31^ underscoring crop- and environment-specific variability. The relatively rapid dissipation observed under our warm field conditions is consistent with temperature-enhanced degradation processes.^32^

Variation in dissipation is attributable to both compound properties and plant–surface effects. Tebufenozide has a higher log *P* (4.25) and vapor pressure (1.56 × 10^−4^ mPa)^33^ produced higher initial deposits, indicative of stronger cuticular affinity, whereas methoxyfenozide (log *P* = 3.72; 1.33 × 10^−6^ mPa)^33^ showed lower initial loading and a steeper early decline. This pattern mirrors established behavior: lipophilic pesticides concentrate on the hydrophobic cuticle, while less lipophilic analogs penetrate tissues or dissipate more rapidly.^15,34^ Environmental drivers (temperature, solar radiation, humidity) further promote volatilization, photolysis, and microbial activity; higher temperatures, in particular, elevate vapor pressure and reaction kinetics, consistent with our open-field findings. The combined influence of substance properties and environmental dynamics explains the observed residue behavior and accords with predictive dissipation frameworks.^35^

### 3.4. Terminal residues

Terminal residues declined steadily across doses and spray regimes. Methoxyfenozide: day-1 residues were 0.6427/0.9144 mg kg^−1^ at 90 g a.i. ha^−1^ (two/three sprays) and 0.8452/1.2136 mg kg^−1^ at 180 g a.i. ha^−1^, falling by day 7 to 0.1401/0.1911 and 0.1886/0.2141 mg kg^−1^ (About 78–82% reduction), all ≤36% of the tomato MRL (0.6 mg kg^−1^). Tebufenozide: day-1 residues were 1.2811/1.1833 mg kg^−1^ at 150 g a.i. ha^−1^ and 2.3557/1.9825 mg kg^−1^ at 300 g a.i. ha^−1^, declining by day 7 to 0.3383/0.5112 and 0.5158/0.8498 mg kg^−1^ (About 57–78% reduction). At 300 g a.i. ha^−1^, day-1 residues exceeded the MRL (1.5 mg kg^−1^), whereas all day-7 residues were below it (about 23–57% of the limit). Overall, higher spray frequency generally yielded higher terminal residues, underscoring the role of application number in residue management.

### 3.5. Risk assessment

Chronic dietary exposure was estimated as the National Estimated Daily Intake (NEDI) using eqn (1) with measured terminal residues from Section 3.4 and the consumption/body-weight defaults (*F* = 0.2009 kg per person per day; bw = 60 kg). Chronic risk was expressed as the Risk Quotient RQ (%) = (NEDI/ADI) × 100 (eqn (2)), adopting ADI = 0.10 mg kg per bw per day for methoxyfenozide and 0.02 mg kg per bw per day for tebufenozide. Across all dosage and spray-frequency scenarios, NEDI values were < ADI, *i.e.*, RQ < 100%, indicating no chronic health concern under the tested GAP and PHIs (Tables 5 and Tables 6).

**Table 5:** Terminal residues, national estimated daily intake (NEDI), and risk quotients (RQ%) of methoxyfenozide in tomato fruits under different application regimes

Dosage (g a.i. ha^−1^)	Number of times sprayed	Days after spraying	Residues (mg kg^−1^)	NEDI (mg per kg bw per day)	RQ%
90	2	1	0.6427	2.15 × 10^−3^	2.15
		3	0.3188	1.07 × 10^−3^	1.07
		7	0.1401	4.69 × 10^−4^	0.47
	3	1	0.9144	3.06 × 10^−3^	3.06
		3	0.4433	1.48 × 10^−3^	1.48
		7	0.1911	6.40 × 10^−4^	0.64
180	2	1	0.8452	2.83 × 10^−3^	2.83
		3	0.4464	1.49 × 10^−3^	1.49
		7	0.1886	6.31 × 10^−4^	0.63
	3	1	1.2136	4.06 × 10^−3^	4.06
		3	0.7124	2.39 × 10^−3^	2.39
		7	0.2141	7.17 × 10^−4^	0.72

**Table 6:** Terminal residues, national estimated daily intake (NEDI), and risk quotients (RQ%) of tebufenozide in tomato fruits under different application regimes

Dosage (g a.i. ha^−1^)	Number of times sprayed	Days after spraying	Residues (mg kg^−1^)	NEDI (mg per kg bw per day)	RQ%
150	2	1	1.2811	4.29 × 10^−3^	21.45
		3	1.0167	3.40 × 10^−3^	17.02
		7	0.3383	1.13 × 10^−3^	5.66
	3	1	1.1833	3.96 × 10^−3^	19.81
		3	0.9188	3.08 × 10^−3^	15.38
		7	0.5158	1.73 × 10^−3^	8.64
300	2	1	2.3557	7.89 × 10^−3^	39.44
		3	1.7768	5.95 × 10^−3^	29.75
		7	0.5112	1.71 × 10^−3^	8.56
	3	1	1.9825	6.64 × 10^−3^	33.19
		3	1.4478	4.85 × 10^−3^	24.24
		7	0.8498	2.85 × 10^−3^	14.23

For methoxyfenozide, the worst-case RQ (%ADI) occurred at 180 g a.i. ha^−1^ with three sprays, Day 1, reaching 4.06%, and declined to ≤0.72% by Day 7. For tebufenozide, the maximum RQ was observed at 300 g a.i. ha^−1^ with two sprays, Day 1, at 39.44%, decreasing to ≤14.23% by Day 7. Thus, even under high-end application programs and at early post-treatment intervals, NEDI remained well below 100% of ADI for both insecticides.

Expressing the results explicitly as %ADI clarifies margin-to-threshold: methoxyfenozide exposures were ≤4.06% ADI and tebufenozide exposures ≤39.44% ADI across all tested scenarios. These findings support the PHIs derived from the dissipation modeling (Section 3.3) and demonstrate that, when GAP and PHIs are respected, long-term dietary risk is acceptable.

### 3.6. Effect of washing treatments

**Fig. 3:**
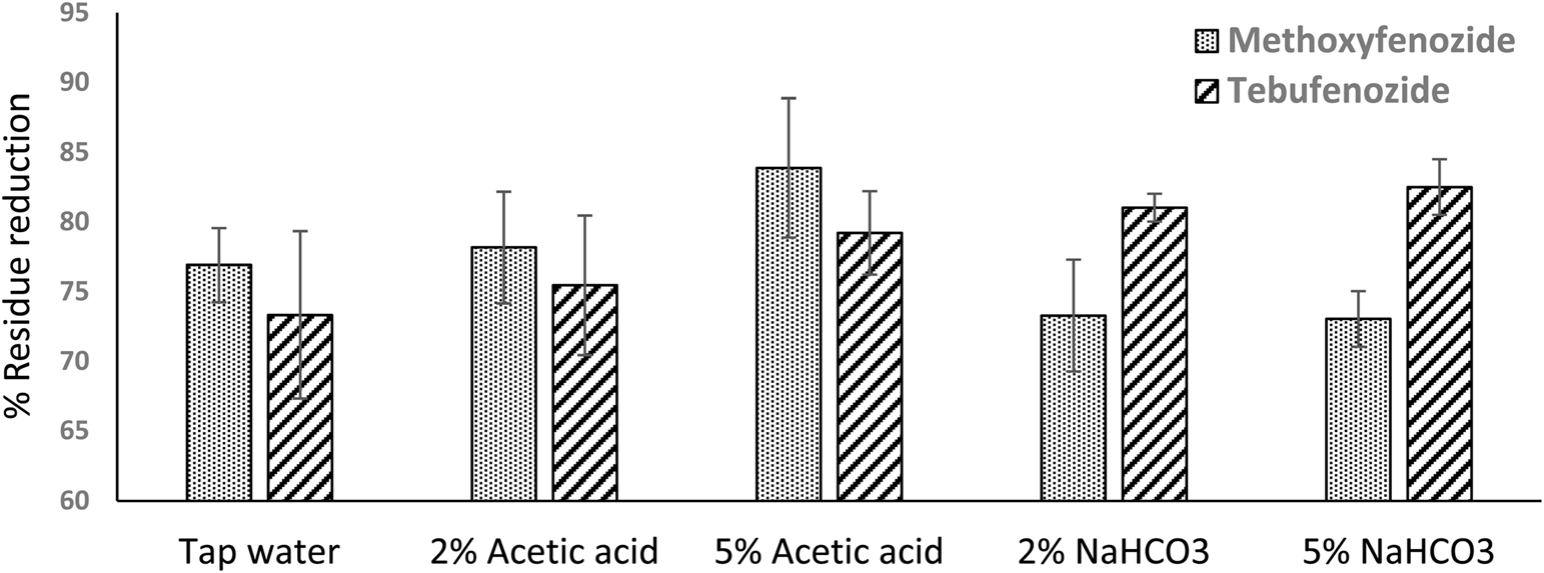
Effect of different washing treatments on the reduction of methoxyfenozide and tebufenozide residues in tomatoes.

Household washing effectively reduced residues of both insecticides (Fig. 3). Tebufenozide removals were 73.33% (tap), 75.45%/79.21% (2%/5% acetic acid), and 81.02%/82.50% (2%/5% NaHCO_3_); among-treatment differences were not significant (one-way ANOVA, *F* = 3.24, *p* = 0.060), consistent with reports that both acidic and alkaline washes lower tomato residues.^36^ Methoxyfenozide removals were 76.91% (tap), 78.17% (2% acetic), 83.87% (5% acetic; highest), and 73.28% (NaHCO_3_), with significant among-treatment effects (*F* = 3.90, *p* = 0.037) and 5% acetic acid outperforming other washes on pairwise tests (*p* < 0.05). Percent removals are referenced to paired, contemporaneous unwashed controls under standardized washing conditions (solution, contact time, temperature, produce-to-solution ratio), and the analytical method confirmed analyte stability with LOQs below all terminal residues. Mechanistically, the patterns align with physicochemical behavior: methoxyfenozide slightly lower lipophilicity and very low vapor pressure render it more responsive to acid-facilitated desorption/hydrolysis, whereas tebufenozide—comparatively acid-stable—shows marginal, non-significant gains with alkali, implying that mechanical rinsing and solution contact dominate its removal.^15,34^ In concert with the dietary-risk assessment, these wash-mediated reductions further lower exposure by day 7; all RQ% remained <100%, indicating acceptable chronic risk under Good Agricultural Practices (GAP).^15,34,36^

## 4. Conclusion

This work establishes a sensitive, reliable, and efficient LC-MS/MS method for the simultaneous determination of methoxyfenozide, tebufenozide, chromafenozide, and halofenozide in tomato fruits, demonstrating excellent linearity, sensitivity, accuracy, and precision suitable for routine monitoring and regulatory compliance. Field dissipation studies under Egyptian conditions showed first-order degradation for methoxyfenozide and tebufenozide with short half-lives and pre-harvest intervals that meet international safety standards; chronic dietary risk assessment indicated exposures well within acceptable limits when good agricultural practices are followed. In addition, simple household processing—particularly washing with 5% acetic acid—substantially reduced residues, offering an accessible means to further minimize dietary exposure. A key limitation is that field trials covered only two compounds in a single climatic region. Future work should evaluate the dissipation of all four insecticides across diverse environments and assess cumulative exposure risks. The findings provide practical evidence to inform regulatory decision-making, strengthen food-safety assurance, and support the sustainable use of insect growth regulators in tomato production.

## Author contributions

Nevein Ahmed and Osama Abdallah contributed equally to the conceptualization, Writing—original draft preparation, supervision, and project administration Rania Abd El-Hamid and Hanim Soliman contributed equally to the formal analysis and investigation, while review and editing was performed by Fahad Almulhim. All authors have read and agreed to the published version of the manuscript.

## Conflicts of interest

The authors declare that they have no known competing financial interests or personal relationships that could have appeared to influence the work reported in this paper.

## Supplementary Material

RA-015-D5RA08001K-s001

## Data Availability

All data supporting this study are available within the article and its electronic Supplementary Information (SI). Supplementary information is available. See DOI: https://doi.org/10.1039/d5ra08001k.
