# Supplementary material for: Simultaneous LC-MS/MS determination of diacylhydrazine ecdysone receptor agonist insecticides in tomatoes: field dissipation, household washing effects, and dietary risk assessment
Source: RSC Adv. 2025 Dec 1;15(55):47220–9. doi: 10.1039/d5ra08001k (PMC12667604; doi:10.1039/d5ra08001k)
Supplement: RA-015-D5RA08001K-s001 [file RA-015-D5RA08001K-s001.pdf]

Supplementary Information (SI) for RSC Advances.
This journal is © The Royal Society of Chemistry 2025

Simultaneous LC–MS/MS determination of diacylhydrazine ecdysone receptor agonist insecticides in tomatoes: field dissipation, household processing effects and dietary risk assessment.

Rania M. Abd El-Hamid[1], Nevein S. Ahmed[1], Hanim M. Soliman[1], Fahad S. Almulhim[2], Osama I. Abdallah[1,2*]

[1]Department of Pesticide Residues and Environmental Pollution, Central Agricultural Pesticide Laboratory, Agricultural Research Center, Giza 12618, Egypt.

[2] Department of Food Chemistry, Food Safety Laboratory, Qassim Municipality, Qassim region, Buraydah,52571, Saudi Arabia.

Corresponding author

E-mail address: shebin_osama@yahoo.com (O.I. Abdallah).

P.O. Box 12618 Giza, Egypt

(a)

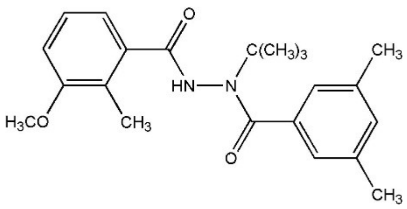

(b)

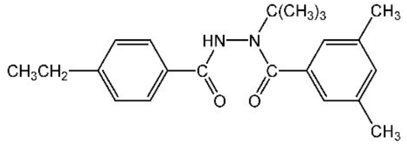

A

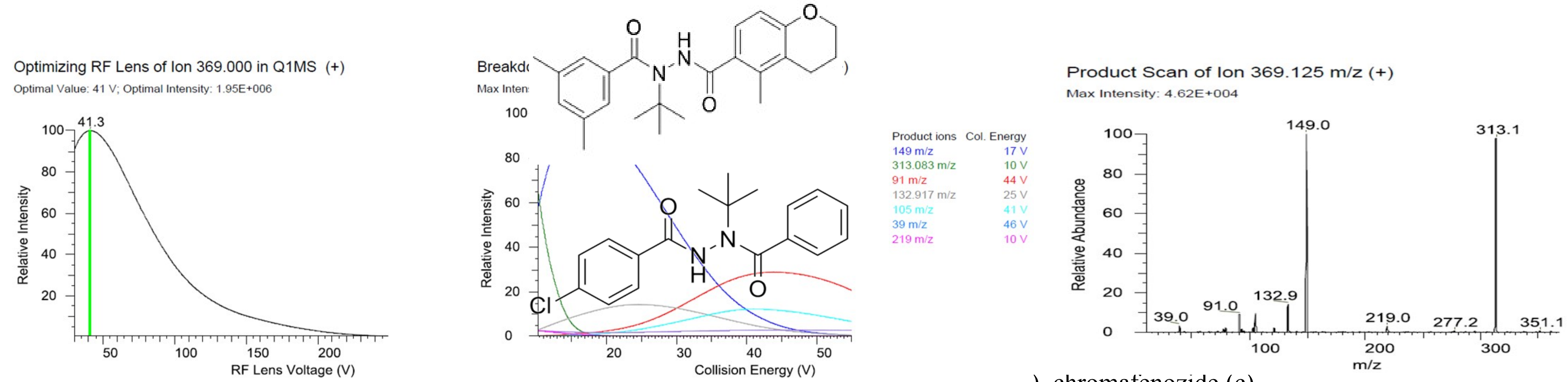

Figure S1. Chemical structure of methoxyfenozide (a), tebufenozide (b), chromafenozide (c),

B

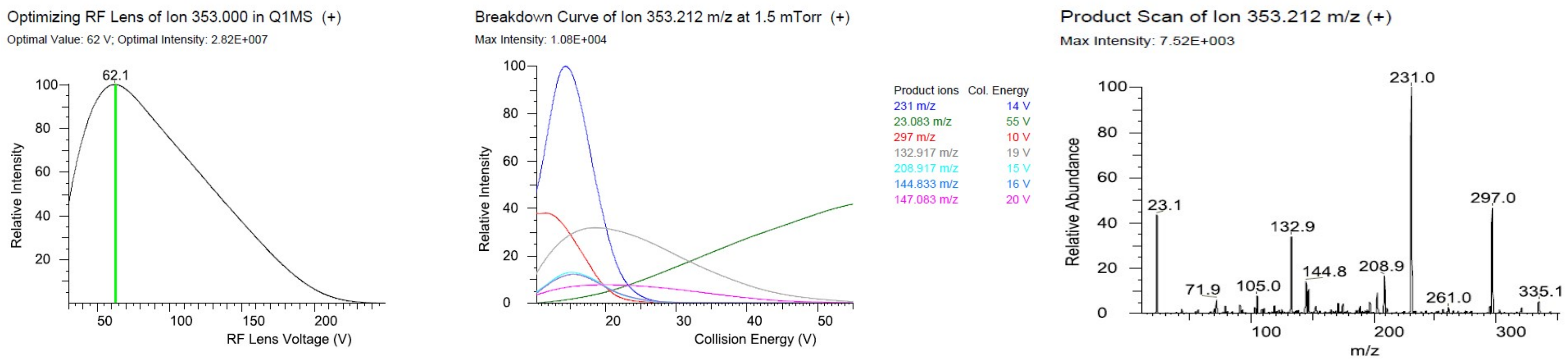

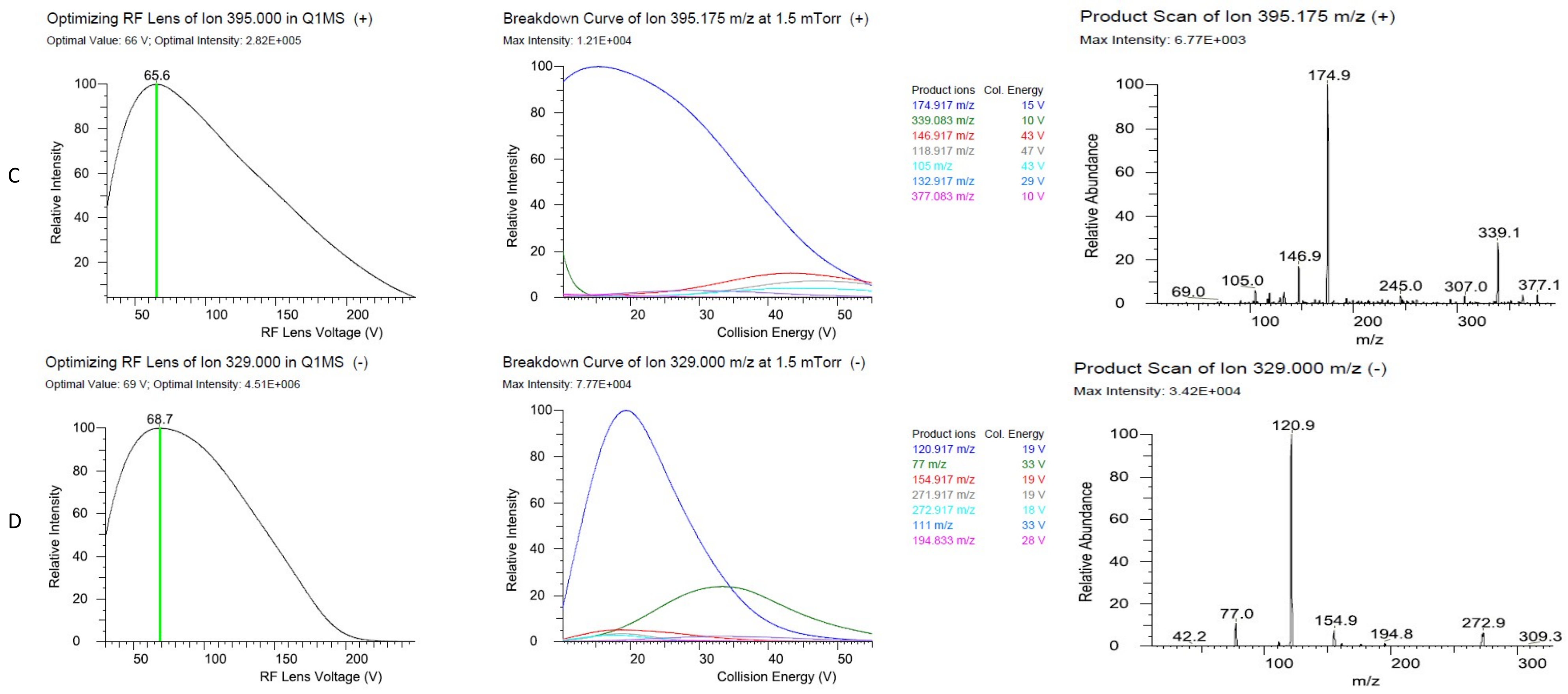

Figure S2. LC–MS/MS optimization and product-ion spectra for (A) methoxyfenozide (ESI$^+$, m/z 369.125), (B) tebufenozide (ESI$^+$, m/z 353.212), (C) chromafenozide (ESI$^+$, m/z 395.175), and (D) halofenozide (ESI$^-$, m/z 329.000). Left: RF-lens tuning; center: collision-energy breakdown curves; right: product-ion scans.

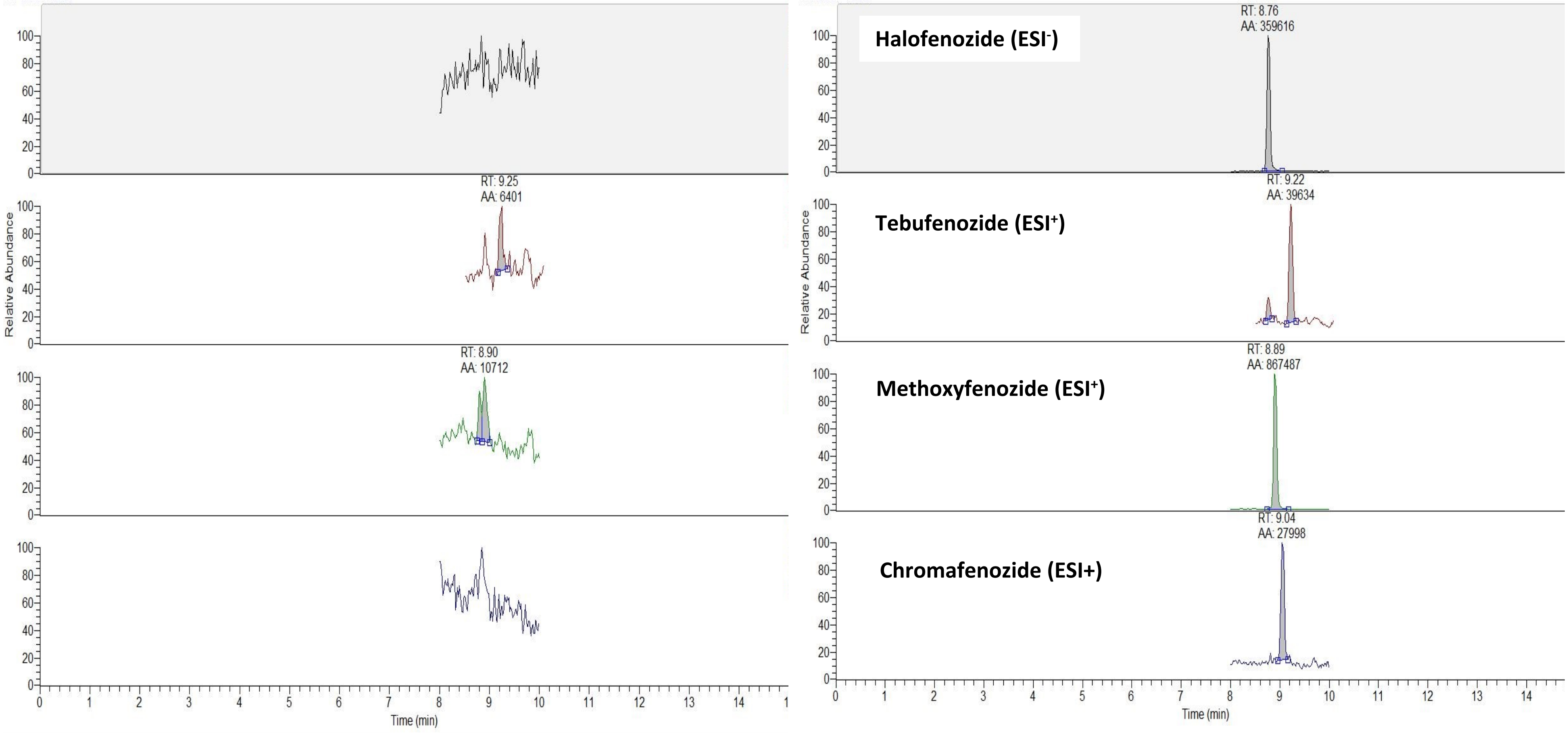

Figure S3. Representative LC–MS/MS chromatograms of blank matrix (left) and matrix spiked at 0.01 mg kg^{-1} (right) for halofenozide (ESI^{-}), tebufenozide (ESI^{+}), methoxyfenozide (ESI^{+}), and chromafenozide (ESI^{+}).

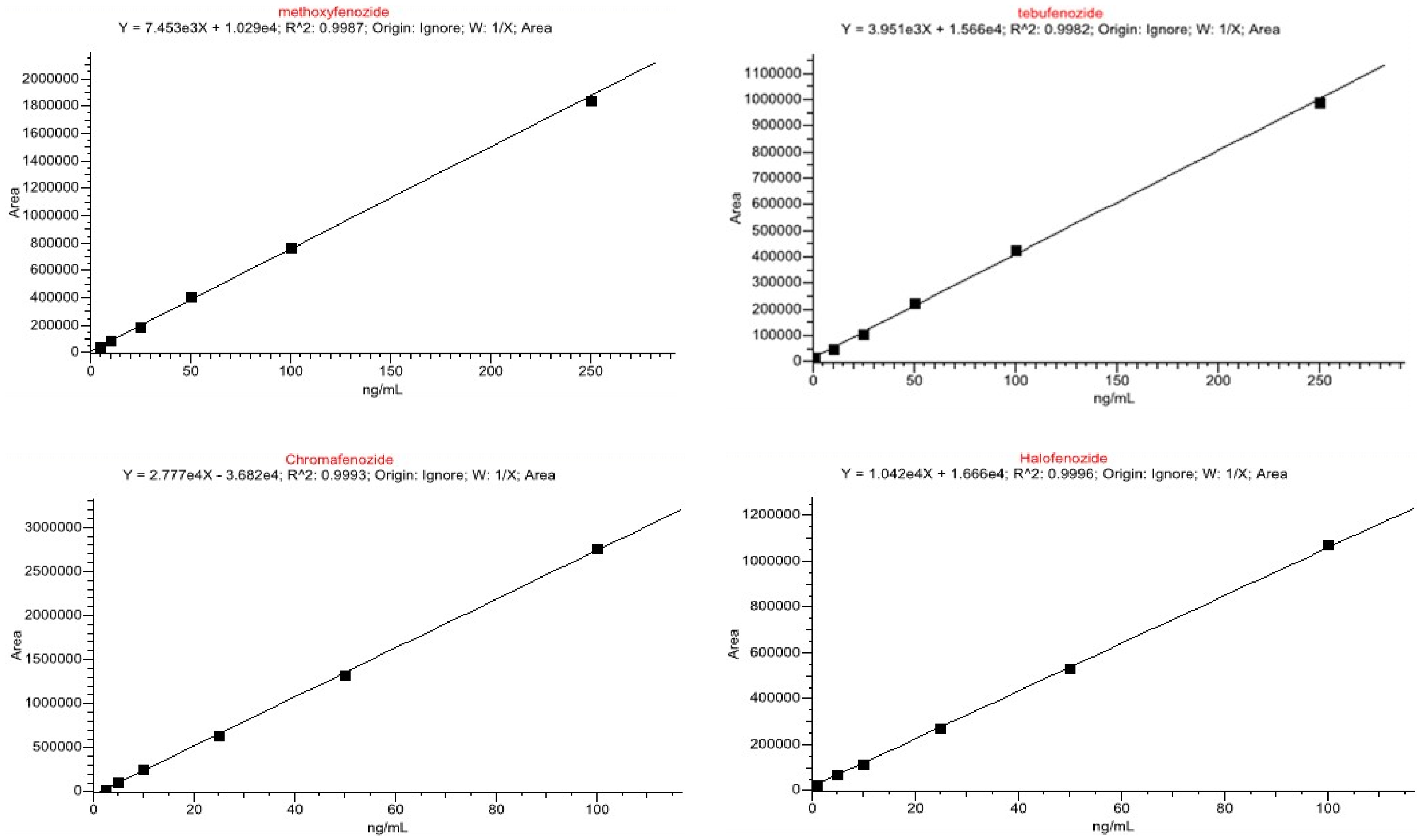

Figure S4. Matrix-matched calibration curves (peak area vs concentration) for methoxyfenozide, tebufenozide, chromafenozide, and halofenozide in blank tomato extract.
